# 1-[5-(4-Meth­oxy­phen­yl)-3-phenyl-4,5-dihydro-1*H*-pyrazol-1-yl]ethanone

**DOI:** 10.1107/S1600536810045861

**Published:** 2010-11-13

**Authors:** Asghar Abbas, Safdar Hussain, Noureen Hafeez, Kong Mun Lo, Aurangzeb Hasan

**Affiliations:** a Department of Chemistry, Quaid-i-Azam University, Islamabad, 45320 Pakistan; b Department of Forensic Medicine & Toxicology, National University of Sciences & Technology, Islamabad, Pakistan; c Department of Chemistry, University of Malaya, 50603 Kuala Lumpur, Malaysia

## Abstract

The title mol­ecule, C_18_H_18_N_2_O_2_, is V-shaped with the pyrazoline moiety being inclined to the adjacent phenyl ring by an angle of 6.49 (9)°, while the 4-meth­oxy-substituted ring is inclined to the pyrazoline ring by 82.99 (9)°. In the crystal, adjacent mol­ecules are linked by C—H⋯O inter­actions, forming chains propagating in [100]. There are also C—H⋯π inter­actions involving adjacent mol­ecules and those related by an inversion center.

## Related literature

For the biological and pharmacological activity of 2-pyrazoline derivatives, see: Hatheway *et al.* (1978 ▶); Lombardino & Ottemes (1981 ▶); Parmar *et al.* (1974 ▶); Rathish *et al.* (2009 ▶); Subbaramaiah *et al.* (2002 ▶). For the synthesis and crystal structure of alk­oxy group-bearing 2-pyrazoline derivatives, see: Abbas *et al.* (2010 ▶); Bai *et al.* (2009 ▶); Lu *et al.* (2008 ▶); Fahrni *et al.* (2003 ▶); Jian *et al.* (2008 ▶).
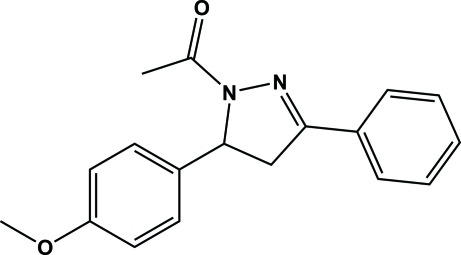


## Experimental

### 

#### Crystal data

C_18_H_18_N_2_O_2_
*M* *_r_* = 294.34
Triclinic, 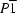

*a* = 6.2762 (9) Å
*b* = 7.2081 (9) Å
*c* = 18.570 (2) Å
α = 85.939 (9)°
β = 85.384 (9)°
γ = 64.709 (8)°
*V* = 756.51 (17) Å^3^
*Z* = 2
Mo *K*α radiation
μ = 0.09 mm^−1^
*T* = 296 K
0.30 × 0.30 × 0.20 mm

#### Data collection

Bruker APEXII CCD area-detector diffractometer
Absorption correction: multi-scan (*SADABS*; Sheldrick, 1996 ▶) *T*_min_ = 0.975, *T*_max_ = 0.983
7199 measured reflections
3448 independent reflections
2584 reflections with *I* > 2σ(*I*)
*R*_int_ = 0.026

#### Refinement

*R*[*F* ^2^ > 2σ(*F* ^2^)] = 0.050
*wR*(*F* ^2^) = 0.164
*S* = 1.08
3448 reflections
201 parameters
H-atom parameters constrained
Δρ_max_ = 0.18 e Å^−3^
Δρ_min_ = −0.26 e Å^−3^

### 

Data collection: *APEX2* (Bruker, 2007 ▶); cell refinement: *SAINT* (Bruker, 2007 ▶); data reduction: *SAINT*; program(s) used to solve structure: *SHELXS97* (Sheldrick, 2008 ▶); program(s) used to refine structure: *SHELXL97* (Sheldrick, 2008 ▶); molecular graphics: *ORTEP-3* (Farrugia, 1997 ▶); software used to prepare material for publication: *publCIF* (Westrip, 2010 ▶).

## Supplementary Material

Crystal structure: contains datablocks I, global. DOI: 10.1107/S1600536810045861/su2223sup1.cif

Structure factors: contains datablocks I. DOI: 10.1107/S1600536810045861/su2223Isup2.hkl

Additional supplementary materials:  crystallographic information; 3D view; checkCIF report

## Figures and Tables

**Table 1:** Hydrogen-bond geometry (Å, °) *Cg1* and *Cg3* are the centroids of the N1,N2,C8–C10 and C11–C16 rings, respectively.

*D*—H⋯*A*	*D*—H	H⋯*A*	*D*⋯*A*	*D*—H⋯*A*
C4—H4⋯O2^i^	0.93	2.47	3.331 (2)	154
C1—H1*C*⋯*Cg*1^ii^	0.96	2.96	3.755 (2)	141
C12—H12⋯*Cg*1^iii^	0.93	2.96	3.7783 (18)	148
C18—H18*A*⋯*Cg*3^iv^	0.96	2.63	3.544 (2)	159

Symmetry codes: (i) 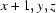; (ii) 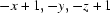; (iii) 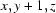; (iv) 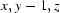.

**Fig. 1.**
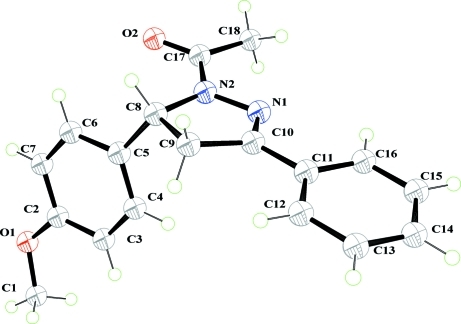
A view of the molecular structure of the title molecule, showing the atom numbering scheme. Displacement ellipsoids are drawn at the 50% probability level.

## supplementary crystallographic information

### Comment

Pyrazoline systems are well known nitrogen-containing heterocyclic compounds which possess a wide range of biological and pharmacological activities such as antitumor (Hatheway *et al.*, 1978), immunosuppressive (Lombardino *et al.*,1981), psychoanaleptic (Parmar *et al.*, 1974), anti-inflammation (Rathish *et al.*, 2009), and anticancer (Subbaramaiah *et al.*, 2002). In continuation of previous structural studies of alkoxy group bearing pyrazoline derivatives (Abbas *et al.*, 2010), the title compound was synthesized and its crystal structure is reported on herein.

The molecular structure of the title compound is shown in Fig. 1. All the bond lengths and bond angles are similar to those observed in similar structures (Fahrni *et al.*, 2003; Bai *et al.*, 2009; Lu *et al.*, 2008). In the pyrazolinyl ring, the C8—N2 and C10=N1 bond lengths, 1.483 (2) and 1.2860 (18) Å, respectively, are comparable with those in similar structures [C—N 1.482 (2)–1.515 (9) A°, C=N 1.291 (2)–1.300 (10) A°]. The N1—N2 bond length of 1.3867 (16) Å is slightly longer than that found in a similar structure [N–N 1.373 (2)–1.380 (8) A°] (Jian *et al.*, 2008). The plane containing the pyrazoline moiety is inclined to the adjacent phenyl ring (C16-C21) by 6.49 (9)\%, while the 4-methoxy substituted phenyl ring (C2-C7) is inclined to the pyrazoline moiety by 82.99 (9) °.

In the crystal adjacent molecules are linked by a C-H···O interaction forming chains propagating in [100]. There are also C-H···π interactions involving adjacent molecules and those related by an inversion center; see Table 1 for details.

### Experimental

To a mixture of *(E)*-3-(4-(methoxy)phenyl)-1-phenylprop-2-en-1-one (2.94 g, 10 mmol) and hydrazine hydrate (1.0 g, 20 mmol) in acetic acid (25 ml), were added two drops of concentrated hydrochloric acid. The mixture was refluxed for 5 h. The precipitated solids were filtered, dried and recrystallized from ethanol. The crystals, suitable for X-ray diffraction analysis, were obtained from a mixture of ethyl acetate and dichloromethane (v:v / 1:1) by slow evaporation.

### Refinement

The H-atoms were placed at calculated positions and were treated as riding: C-H = 0.93, 0.96, 0.97 and 0.98 Å for CH(aromatic), methylene, methyl and methine H-atoms, respectively, with U_iso_(H) = k × *U*_eq_(C), where k = 1.5 for methyl H-atoms and 1.2 for all other H-atoms.

### Crystal data

**Table:**

C_18_H_18_N_2_O_2_	*Z* = 2
*M**_r_* = 294.34	*F*(000) = 312
Triclinic, *P*1	*D*_x_ = 1.292 Mg m^−^^3^
Hall symbol: -P 1	Mo *K*α radiation, λ = 0.71073 Å
*a* = 6.2762 (9) Å	Cell parameters from 2249 reflections
*b* = 7.2081 (9) Å	θ = 3.1–26.2°
*c* = 18.570 (2) Å	µ = 0.09 mm^−^^1^
α = 85.939 (9)°	*T* = 296 K
β = 85.384 (9)°	Block, white
γ = 64.709 (8)°	0.30 × 0.30 × 0.20 mm
*V* = 756.51 (17) Å^3^	

### Data collection

**Table:**

Bruker APEXII CCD area-detector diffractometer	3448 independent reflections
Radiation source: fine-focus sealed tube	2584 reflections with *I* > 2σ(*I*)
graphite	*R*_int_ = 0.026
ω scans	θ_max_ = 27.5°, θ_min_ = 1.1°
Absorption correction: multi-scan (*SADABS*; Sheldrick, 1996)	*h* = −8→7
*T*_min_ = 0.975, *T*_max_ = 0.983	*k* = −9→9
7199 measured reflections	*l* = −24→24

### Refinement

**Table:**

Refinement on *F*^2^	Primary atom site location: structure-invariant direct methods
Least-squares matrix: full	Secondary atom site location: difference Fourier map
*R*[*F*^2^ > 2σ(*F*^2^)] = 0.050	Hydrogen site location: inferred from neighbouring sites
*wR*(*F*^2^) = 0.164	H-atom parameters constrained
*S* = 1.08	*w* = 1/[σ^2^(*F*_o_^2^) + (0.0912*P*)^2^ + 0.0545*P*] where *P* = (*F*_o_^2^ + 2*F*_c_^2^)/3
3448 reflections	(Δ/σ)_max_ < 0.001
201 parameters	Δρ_max_ = 0.18 e Å^−^^3^
0 restraints	Δρ_min_ = −0.26 e Å^−^^3^

### Special details

**Table:**

Geometry. All e.s.d.'s (except the e.s.d. in the dihedral angle between two l.s. planes) are estimated using the full covariance matrix. The cell e.s.d.'s are taken into account individually in the estimation of e.s.d.'s in distances, angles and torsion angles; correlations between e.s.d.'s in cell parameters are only used when they are defined by crystal symmetry. An approximate (isotropic) treatment of cell e.s.d.'s is used for estimating e.s.d.'s involving l.s. planes.
Refinement. Refinement of *F*^2^ against ALL reflections. The weighted *R*-factor *wR* and goodness of fit *S* are based on *F*^2^, conventional *R*-factors *R* are based on *F*, with *F* set to zero for negative *F*^2^. The threshold expression of *F*^2^ > σ(*F*^2^) is used only for calculating *R*-factors(gt) *etc*. and is not relevant to the choice of reflections for refinement. *R*-factors based on *F*^2^ are statistically about twice as large as those based on *F*, and *R*- factors based on ALL data will be even larger.

### Fractional atomic coordinates and isotropic or equivalent isotropic displacement parameters (Å^2^)

**Table:**

	*x*	*y*	*z*	*U*_iso_*/*U*_eq_	
O1	0.4631 (3)	−0.2736 (2)	0.45861 (9)	0.0843 (5)	
C3	0.4986 (3)	−0.0139 (3)	0.37307 (9)	0.0537 (4)	
H3	0.6618	−0.0701	0.3751	0.064*	
C4	0.3831 (3)	0.1595 (3)	0.32925 (9)	0.0500 (4)	
H4	0.4709	0.2180	0.3018	0.060*	
O2	−0.1965 (2)	0.2057 (2)	0.21830 (7)	0.0643 (4)	
C16	0.4222 (3)	0.6534 (2)	0.08234 (9)	0.0494 (4)	
H16	0.3703	0.5800	0.0546	0.059*	
N2	0.0349 (2)	0.3699 (2)	0.20010 (7)	0.0452 (3)	
N1	0.1673 (2)	0.44578 (18)	0.15426 (7)	0.0401 (3)	
C1	0.7049 (4)	−0.3530 (3)	0.47300 (13)	0.0760 (6)	
H1A	0.7996	−0.3994	0.4291	0.114*	
H1B	0.7424	−0.4661	0.5077	0.114*	
H1C	0.7366	−0.2475	0.4920	0.114*	
C2	0.3689 (3)	−0.1025 (3)	0.41372 (9)	0.0564 (4)	
C5	0.1405 (3)	0.2471 (2)	0.32553 (8)	0.0456 (4)	
C8	0.0129 (3)	0.4317 (2)	0.27581 (8)	0.0472 (4)	
H8	−0.1543	0.4988	0.2920	0.057*	
C9	0.1164 (3)	0.5910 (3)	0.26675 (9)	0.0511 (4)	
H9A	−0.0054	0.7288	0.2738	0.061*	
H9B	0.2381	0.5615	0.3005	0.061*	
C10	0.2176 (2)	0.5667 (2)	0.19012 (8)	0.0388 (3)	
C11	0.3582 (3)	0.6705 (2)	0.15576 (8)	0.0399 (3)	
C15	0.5613 (3)	0.7436 (3)	0.05020 (10)	0.0601 (5)	
H15	0.6047	0.7297	0.0011	0.072*	
C14	0.6364 (3)	0.8549 (3)	0.09092 (11)	0.0597 (5)	
H14	0.7331	0.9138	0.0694	0.072*	
C13	0.5693 (3)	0.8791 (3)	0.16288 (10)	0.0558 (4)	
H13	0.6178	0.9566	0.1899	0.067*	
C12	0.4290 (3)	0.7881 (2)	0.19563 (9)	0.0481 (4)	
H12	0.3823	0.8059	0.2444	0.058*	
C17	−0.0737 (3)	0.2610 (2)	0.17608 (9)	0.0460 (4)	
C18	−0.0336 (3)	0.2129 (3)	0.09780 (10)	0.0546 (4)	
H18A	0.1118	0.0928	0.0906	0.082*	
H18B	−0.0251	0.3269	0.0697	0.082*	
H18C	−0.1617	0.1882	0.0828	0.082*	
C6	0.0143 (3)	0.1551 (3)	0.36706 (9)	0.0538 (4)	
H6	−0.1492	0.2116	0.3657	0.065*	
C7	0.1271 (3)	−0.0172 (3)	0.40986 (10)	0.0599 (5)	
H7	0.0396	−0.0774	0.4366	0.072*	

### Atomic displacement parameters (Å^2^)

**Table:**

	*U*^11^	*U*^22^	*U*^33^	*U*^12^	*U*^13^	*U*^23^
O1	0.0905 (11)	0.0808 (10)	0.0967 (11)	−0.0527 (9)	−0.0259 (9)	0.0337 (8)
C3	0.0521 (9)	0.0614 (10)	0.0550 (10)	−0.0316 (8)	0.0020 (7)	−0.0057 (8)
C4	0.0551 (9)	0.0630 (10)	0.0458 (8)	−0.0396 (8)	0.0082 (7)	−0.0047 (7)
O2	0.0651 (8)	0.0827 (9)	0.0682 (8)	−0.0548 (7)	0.0016 (6)	0.0011 (7)
C16	0.0627 (10)	0.0455 (8)	0.0521 (9)	−0.0349 (8)	0.0060 (7)	−0.0092 (7)
N2	0.0524 (8)	0.0529 (7)	0.0433 (7)	−0.0356 (6)	0.0019 (6)	−0.0019 (6)
N1	0.0424 (6)	0.0418 (6)	0.0435 (7)	−0.0257 (5)	−0.0004 (5)	0.0006 (5)
C1	0.0838 (15)	0.0640 (12)	0.0780 (14)	−0.0283 (11)	−0.0145 (11)	0.0035 (10)
C2	0.0732 (12)	0.0592 (10)	0.0508 (9)	−0.0416 (9)	−0.0063 (8)	0.0018 (8)
C5	0.0541 (9)	0.0554 (9)	0.0390 (7)	−0.0353 (8)	0.0072 (6)	−0.0070 (6)
C8	0.0514 (9)	0.0537 (9)	0.0443 (8)	−0.0307 (7)	0.0068 (7)	−0.0067 (7)
C9	0.0660 (10)	0.0497 (9)	0.0473 (9)	−0.0343 (8)	0.0059 (7)	−0.0084 (7)
C10	0.0407 (7)	0.0358 (7)	0.0438 (8)	−0.0201 (6)	−0.0016 (6)	−0.0021 (6)
C11	0.0418 (7)	0.0324 (7)	0.0492 (8)	−0.0194 (6)	−0.0034 (6)	0.0003 (6)
C15	0.0774 (12)	0.0567 (10)	0.0590 (10)	−0.0433 (9)	0.0167 (9)	−0.0087 (8)
C14	0.0634 (11)	0.0516 (10)	0.0783 (13)	−0.0400 (9)	0.0057 (9)	−0.0007 (9)
C13	0.0650 (11)	0.0491 (9)	0.0698 (11)	−0.0387 (8)	−0.0122 (9)	−0.0011 (8)
C12	0.0571 (9)	0.0458 (8)	0.0504 (9)	−0.0297 (7)	−0.0062 (7)	−0.0014 (7)
C17	0.0424 (8)	0.0482 (8)	0.0566 (9)	−0.0282 (7)	−0.0051 (7)	0.0034 (7)
C18	0.0631 (10)	0.0573 (10)	0.0587 (10)	−0.0387 (9)	−0.0103 (8)	−0.0033 (8)
C6	0.0554 (10)	0.0712 (11)	0.0497 (9)	−0.0426 (9)	0.0052 (7)	−0.0016 (8)
C7	0.0717 (12)	0.0772 (12)	0.0520 (9)	−0.0541 (10)	0.0013 (8)	0.0071 (8)

### Geometric parameters (Å, °)

**Table:**

O1—C2	1.370 (2)	C8—C9	1.537 (2)
O1—C1	1.415 (2)	C8—H8	0.9800
C3—C2	1.384 (2)	C9—C10	1.501 (2)
C3—C4	1.388 (2)	C9—H9A	0.9700
C3—H3	0.9300	C9—H9B	0.9700
C4—C5	1.382 (2)	C10—C11	1.467 (2)
C4—H4	0.9300	C11—C12	1.389 (2)
O2—C17	1.2202 (19)	C15—C14	1.380 (3)
C16—C15	1.374 (2)	C15—H15	0.9300
C16—C11	1.389 (2)	C14—C13	1.369 (3)
C16—H16	0.9300	C14—H14	0.9300
N2—C17	1.3544 (19)	C13—C12	1.388 (2)
N2—N1	1.3867 (16)	C13—H13	0.9300
N2—C8	1.483 (2)	C12—H12	0.9300
N1—C10	1.2860 (18)	C17—C18	1.495 (2)
C1—H1A	0.9600	C18—H18A	0.9600
C1—H1B	0.9600	C18—H18B	0.9600
C1—H1C	0.9600	C18—H18C	0.9600
C2—C7	1.379 (3)	C6—C7	1.370 (3)
C5—C6	1.393 (2)	C6—H6	0.9300
C5—C8	1.516 (2)	C7—H7	0.9300
			
C2—O1—C1	118.98 (16)	C8—C9—H9B	111.2
C2—C3—C4	119.52 (16)	H9A—C9—H9B	109.1
C2—C3—H3	120.2	N1—C10—C11	120.66 (14)
C4—C3—H3	120.2	N1—C10—C9	113.99 (13)
C5—C4—C3	121.48 (15)	C11—C10—C9	125.34 (13)
C5—C4—H4	119.3	C16—C11—C12	118.70 (14)
C3—C4—H4	119.3	C16—C11—C10	120.40 (13)
C15—C16—C11	120.83 (15)	C12—C11—C10	120.90 (14)
C15—C16—H16	119.6	C16—C15—C14	119.84 (17)
C11—C16—H16	119.6	C16—C15—H15	120.1
C17—N2—N1	122.42 (13)	C14—C15—H15	120.1
C17—N2—C8	124.22 (13)	C13—C14—C15	120.30 (15)
N1—N2—C8	113.24 (11)	C13—C14—H14	119.8
C10—N1—N2	108.02 (12)	C15—C14—H14	119.8
O1—C1—H1A	109.5	C14—C13—C12	120.07 (15)
O1—C1—H1B	109.5	C14—C13—H13	120.0
H1A—C1—H1B	109.5	C12—C13—H13	120.0
O1—C1—H1C	109.5	C13—C12—C11	120.19 (16)
H1A—C1—H1C	109.5	C13—C12—H12	119.9
H1B—C1—H1C	109.5	C11—C12—H12	119.9
O1—C2—C7	115.83 (16)	O2—C17—N2	119.62 (16)
O1—C2—C3	124.75 (17)	O2—C17—C18	123.10 (14)
C7—C2—C3	119.42 (16)	N2—C17—C18	117.28 (13)
C4—C5—C6	117.83 (15)	C17—C18—H18A	109.5
C4—C5—C8	122.07 (14)	C17—C18—H18B	109.5
C6—C5—C8	120.04 (15)	H18A—C18—H18B	109.5
N2—C8—C5	110.93 (13)	C17—C18—H18C	109.5
N2—C8—C9	100.75 (12)	H18A—C18—H18C	109.5
C5—C8—C9	115.68 (13)	H18B—C18—H18C	109.5
N2—C8—H8	109.7	C7—C6—C5	121.05 (16)
C5—C8—H8	109.7	C7—C6—H6	119.5
C9—C8—H8	109.7	C5—C6—H6	119.5
C10—C9—C8	102.96 (12)	C6—C7—C2	120.69 (16)
C10—C9—H9A	111.2	C6—C7—H7	119.7
C8—C9—H9A	111.2	C2—C7—H7	119.7
C10—C9—H9B	111.2		
			
C2—C3—C4—C5	0.4 (3)	C8—C9—C10—C11	−173.86 (14)
C17—N2—N1—C10	170.50 (14)	C15—C16—C11—C12	2.7 (2)
C8—N2—N1—C10	−5.73 (17)	C15—C16—C11—C10	−177.55 (15)
C1—O1—C2—C7	−171.49 (18)	N1—C10—C11—C16	5.5 (2)
C1—O1—C2—C3	8.9 (3)	C9—C10—C11—C16	−173.24 (14)
C4—C3—C2—O1	179.81 (17)	N1—C10—C11—C12	−174.79 (13)
C4—C3—C2—C7	0.2 (3)	C9—C10—C11—C12	6.5 (2)
C3—C4—C5—C6	−0.3 (2)	C11—C16—C15—C14	−0.8 (3)
C3—C4—C5—C8	−177.65 (15)	C16—C15—C14—C13	−1.2 (3)
C17—N2—C8—C5	70.55 (18)	C15—C14—C13—C12	1.3 (3)
N1—N2—C8—C5	−113.30 (14)	C14—C13—C12—C11	0.7 (3)
C17—N2—C8—C9	−166.43 (15)	C16—C11—C12—C13	−2.7 (2)
N1—N2—C8—C9	9.72 (16)	C10—C11—C12—C13	177.64 (14)
C4—C5—C8—N2	77.68 (18)	N1—N2—C17—O2	−176.77 (14)
C6—C5—C8—N2	−99.57 (17)	C8—N2—C17—O2	−1.0 (2)
C4—C5—C8—C9	−36.3 (2)	N1—N2—C17—C18	3.8 (2)
C6—C5—C8—C9	146.50 (15)	C8—N2—C17—C18	179.57 (14)
N2—C8—C9—C10	−9.34 (15)	C4—C5—C6—C7	−0.3 (3)
C5—C8—C9—C10	110.31 (14)	C8—C5—C6—C7	177.01 (16)
N2—N1—C10—C11	179.75 (12)	C5—C6—C7—C2	1.0 (3)
N2—N1—C10—C9	−1.36 (17)	O1—C2—C7—C6	179.48 (17)
C8—C9—C10—N1	7.31 (17)	C3—C2—C7—C6	−0.8 (3)

### Hydrogen-bond geometry (Å, °)

**Table:**

*Cg1* and *Cg3* are the centroids of the N1,N2,C8–C10 and C11–C16 rings, respectively.

**Table:**

*D*—H···*A*	*D*—H	H···*A*	*D*···*A*	*D*—H···*A*
C4—H4···O2^i^	0.93	2.47	3.331 (2)	154
C1—H1C···Cg1^ii^	0.96	2.96	3.755 (2)	141
C12—H12···Cg1^iii^	0.93	2.96	3.7783 (18)	148
C18—H18A···Cg3^iv^	0.96	2.63	3.544 (2)	159

Symmetry codes: (i) *x*+1, *y*, *z*; (ii) −*x*+1, −*y*, −*z*+1; (iii) *x*, *y*+1, *z*; (iv) *x*, *y*−1, *z*.
